# A Novel Isoquinoline Derivative Anticancer Agent and Its Targeted Delivery to Tumor Cells Using Transferrin-Conjugated Liposomes

**DOI:** 10.1371/journal.pone.0136649

**Published:** 2015-08-26

**Authors:** Xuewei Yang, Shuang Yang, Hongyu Chai, Zhaogang Yang, Robert J. Lee, Weiwei Liao, Lesheng Teng

**Affiliations:** 1 College of Life Sciences, Jilin University, Changchun, China; 2 College of Pharmacy, The Ohio State University, Columbus, United States of America; 3 Department of Organic Chemistry, College of Chemistry, Jilin University, Changchun, China; Okayama University, JAPAN

## Abstract

We have screened 11 isoquinoline derivatives and α-methylene-γ-butyrolactones using the 3-(4,5-dimethylthi-azol-2-yl)-2,5-diphenyltetrazolium bromide (MTT) cytotoxicity assay in HeLa and HEK-293T cells. Compound 2 was identified as potential anticancer agent. To further improve its therapeutic potential, this agent was incorporated into transferrin (Tf)-conjugated liposomes (LPs) for targeted delivery to tumor cells. We have demonstrated Tf-LP-Compound 2 have superior antitumor activity compared to non-targeted controls and the free drug. These data show Tf-LP-Compound 2 to be a promising agent that warrants further evaluation.

## Introduction

Isoquinoline derivatives and α-methylene-γ-butyrolactones are abundant in natural products. They have been identified as bioactive ingredients in many natural product-based therapeutics [1–3]. Current chemotherapies for cancer are mostly cytotoxics with serious side effects and high incidence of drug resistance. New agents with increased efficacy and reduced toxicity are needed. We have synthesized a series of isoquinoline derivatives and α-methylene-γ-butyrolactones [4, 5]. These compounds have shown both excellent stability and significant anticancer activity [6–8]. However, they have poor solubility and their activity is moderate. We believe delivery of these compounds in tumor-cell targeted liposomes (LPs) will address both the solubility problem and facilitate their selective delivery into tumor cells.

LPs can be used as carriers for genes, chemotherapeutic drugs and other therapeutics. Coating of LPs with PEG can extend their circulation half-life [9–13]. Transferrin (Tf) receptor is frequently over-expressed on tumor cells. Human Tf has been used previously for targeting LPs because of its ability to undergo receptor-mediated endocytosis [14–19].

In this study, MTT assay was used to select compounds with superior anti-tumor activity. Compound 2 was selected and encapsulated into LPs conjugated to Tf. Cellular internalization of the LPs was examined by flow cytometry and confocal microscopy [20–22].

## Experimental

### Materials

Hydrogenated soy phosphatidylcholine (HSPC), cholesterol (Chol), methoxy-poly (ethylene glycol) 2000-distearoyl phosphatidylethanolamine (PEG-DSPE) were purchased from Lipoid (Ludwigshafen, Germany).3-(4, 5-Dimethylthiazol-2-yl)-2,5-diphenyltetrazolium bromide (MTT) was obtained from Sigma-Aldrich (St. Louis, MO). Fetal bovine serum (FBS) was obtained from Gibco (Gibco BRL Co. Ltd, USA). DCFH-DA, a fluorescent dye, was obtained from Nanjing Jiancheng Bioengineering Institute (Nanjing, China). 5, 5′, 6, 6′-Tetrachloro-1, 1′, 3, 3′-tetraethyl-imidacarbocyanine iodide (JC-1), 1, 2-dioleoyl-sn-glycero-3-phosphoethanolamine, 7-nitrobenzofurazan-labeled (NBD-DOPE), sulforhodamine B, and holo human Tf were purchased from Sigma-Aldrich (St. Louis, MO). 4', 6-Diamidino-2-phenylindole (DAPI) was purchased from Invitrogen Molecular Probes (Oregon). HeLa, HepG2, HEK-293T cell lines were purchased from American Type Culture Collection (ATCC).

### Cell culture

HeLa, HepG2 and HEK-293T cell lines were grown and propagated in Dulbecco’s modified Eagle’s medium (DMEM), supplemented with 10% FBS at 37°C and humidified atmosphere containing 5% CO_2_.

### MTT screening assay

**Fig 1:**
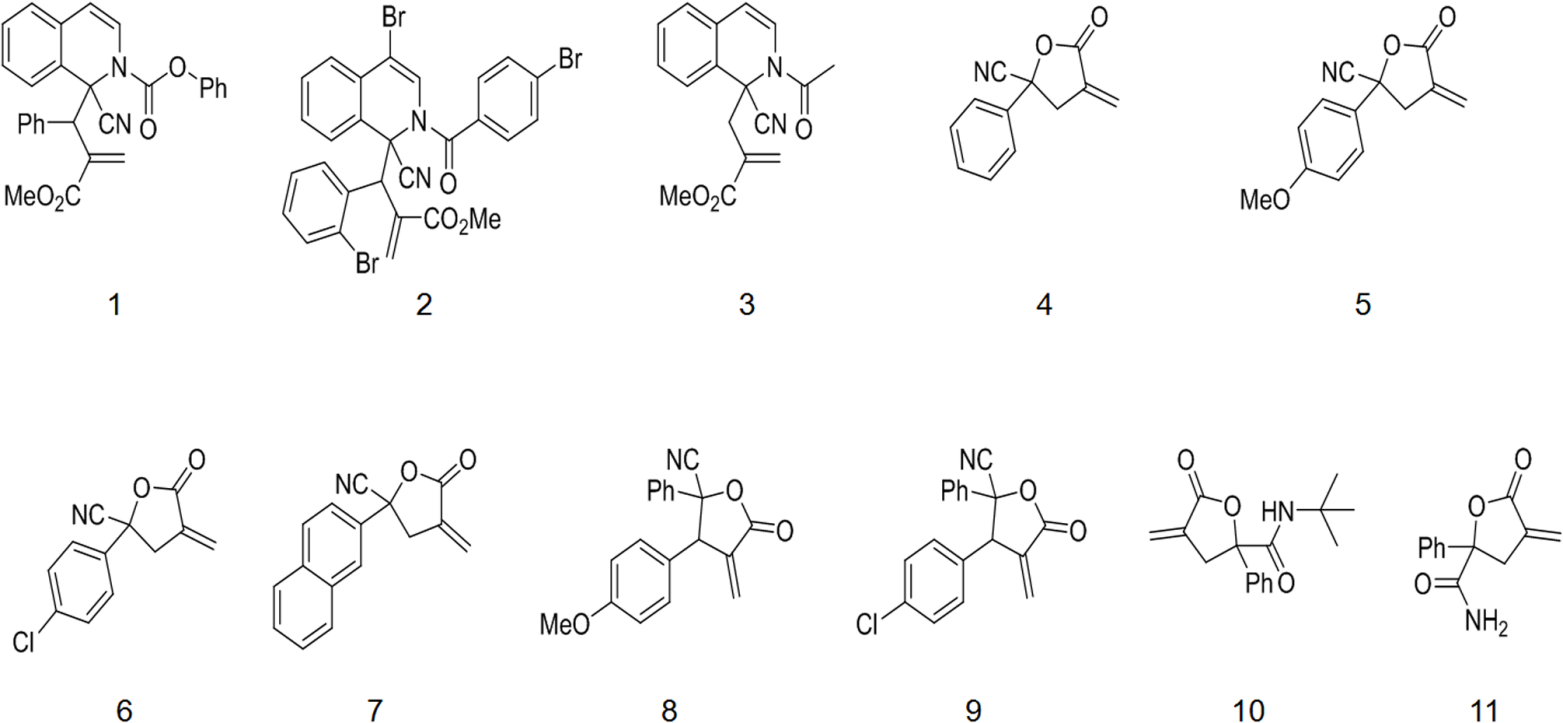
Chemical structure of test compounds.

A total of 11 compounds (Fig 1) were evaluated in HeLa HepG2, and HEK-293T cells, using the MTT assay. Cells were seeded at a density of 1×10^4^ cells/well in a 96-well plate and grown for another 24 h. HeLa and HepG2 cells were then incubated with various compound-containing formulations. HEK-293T cells were incubated with the compounds at 1.5ng/100μl per well. Plates were incubated at 37°C for 4 h and another 100 μl of fresh medium was added to each well. Cells were incubated for an additional 20 h. Then, 10μl of MTT stock solution(5mg/ml) was added to each well, which was incubated at 37°C for 4 h. Absorbance was recorded at 490 nm by a microplate reader (Synergy4, multi-mode microplate reader, BioTek, Winooski, VT, USA).

### Preparation of LPs

Compound 2 has poor solubility. LPs encapsulating Compound 2 were prepared by an ethanol dilution method. Briefly, lipids HSPC, Chol and PEG-DSPE were combined at the at 65:34.5:0.5 (mol%);0.3μmol Compound 2 was added to 6.0μmol lipid mixture. Then the mixture (10 mg/ml) was slowly added to PBS (pH7.4) under vortexing to achieve a final ethanol concentration of 10%. Then, the solution was sonicated with a bath-type sonicator for 30s. The preparation was then passed through a syringe filter (Pall Corporation) to remove unentrapped Compound 2.

For fluorescence labeling of the lipid membrane, NBD-DOPE (1 mole% of total lipids) was added to the lipid mixture [23]. In addition, double-labeled LPs were prepared using 10 mM sulforhodamine B for fluorescence labelling of the aqueous phase. In order to remove free sulforhodamine B, LPs were placed in ultrafiltration centrifuge tube (MWCO 10,000) at the top of the cannula and centrifuged in 10,000rpm for 30min at 4°C. Then the LPs in the ultrafiltration centrifuge tube were suspended with PBS solution and stored in the dark at 4°C. For preparation of Tf conjugated LPs, a post-insertion method was used. Tf-PEG-DSPE was synthesized as described previously [24]. For post-insertion, LPs were incubated with Tf-PEG-DSPE at 37°C for 30 min. Varying concentrations of Tf-PEG-DSPE were incubated with LPs or fluorescence-labeled LPs, and the uptake efficiency of the Tf-conjugated LPs was determined by BCA assay and by flow cytometry.

### Liposomal characterization

Mean particle size and zeta potential of the LPs were determined by Zetasizer Nano ZS 90 (Malvern Instruments, Ltd., Malvern, UK). In addition, the size and surface morphology of the Tf-LP-Compound 2 was investigated by field emission scanning electron microscope (FE-SEM) (JSM-6700F, JEOL, Japan). The sample was fixed on a brass stub using double-sided adhesive tape and was coated with a thin layer of gold and then the images were taken at 3.0kV accelerating voltage.2.6

### In-vitro drug release

Rates of in vitro release of Compound 2 encapsulated in LP and Tf-LP were determined[25]. Liposomal solutions (0.5 mL) were placed in dialysis bag and immersed in 20 mL of release medium (PBS containing 0.5% Tween 80), under 100 rpm magnetic stirring at 37°C. At predetermined time intervals, 1 mL samples were withdrawn and immediately replaced with an equal volume of the fresh medium. The concentration of Compound 2 in the samples was measured on a microplate reader. The cumulative percentage of drug release was calculated and plotted versus time.

### Cytotoxicity assay

Cells were seeded at a density of 1×10^4^ cells/well in a 96-well plate and grown for another 24 h. Cells were then incubated with Compound 2, LP with Compound 2, or Tf-LP with Compound 2. Plates were incubated at 37°C for 4 h and another 100 μL of fresh medium was added to each well. Cells were incubated for an additional 1, 2, 4, 12, 24, 48h. Then, 10μLof MTT solution (5mg/ml) was added to each well. The cells were incubated at 37°C for 4 h. Absorbance was recorded at 490 nm by a microplate reader (Synergy4, multi-mode microplate reader, BioTek, Winooski, VT, USA).

### Mitochondrial transmembrane potential (ΔΨm) assay

JC-1 is a fluorescent probe that is sensitive to mitochondrial membrane potential (ΔΨm)[26, 27]. At high mitochondrial membrane potential, JC-1 concentrates in the mitochondrial matrix to form J-aggregates, which emit red fluorescence. At low mitochondrial membrane potential, JC-1 cannot concentrate in the mitochondria matrix, the JC-1 monomer (monomer) produces green fluorescence. The relative proportion of red and green fluorescence is commonly used to measure the degree of mitochondrial depolarization. A decrease in red/green ratio is indicative of apoptosis. The cells were grown in 6-well plate and treated with Compound 2, LP-Compound 2 and Tf-LP-Compound 2. After 12h exposure, the cells were washed with PBS and incubated with 2 μM of JC-1 dye in PBS (pH7.4) at 37°C in the dark for 20 min. The images were then taken by inverted fluorescent microscope and the mitochondrial depolarization patterns of cells for quantification were examined using imaging software Image J.

### Uptake of LP and Tf-LP by HeLa cells

Cellular uptake of LPs was determined using methods similar to those published previously [28, 29]. Cells (1×10^5^) were seeded in a 24-well plate and cultured for 24h. Then culture medium was replaced with free Compound 2, LP-Compound 2, or Tf-LP-Compound 2 diluted in fresh culture medium and cultured for 4h. The cells were then washed three times with phosphate-buffered saline (PBS, pH 7.4) and fixed with 350 μl 4% formaldehyde solutions. The percentages of positive cells with NBD-DOPE and sulforhodamine B was analyzed by EPICS XL flow cytometer (Beckman Coulter Corp., Brea, CA, USA) and the data were analyzed with the Cell Quest software. Ten thousand cells were counted in triplicate for each cell type.

### Confocal microscopy and cellular internalization analysis

Cells (1.5× 10^5^) were seeded in a 35mm glass bottom culture dishes and cultured for 24h. Then culture medium was replaced with free Compound 2, LP-Compound 2, or Tf-LP-Compound 2 diluted in fresh culture medium and cultured for 4h. After the incubation, the cells were washed three times with phosphate-buffered saline (PBS, pH 7.4) and fixed with 400 μl 4% formaldehyde for 8 min. The cells were then washed. Cellular nuclei were stained with DAPI for 3 minutes, and then cells were washed again. After replacement of PBS, the cells were immediately examined by a Zeiss 710 LSMNLO Confocal Microscope (Carl Zeiss; Jena, Germany)[30].

### Statistical analysis

The data were analyzed for statistical significance Student’s t-test and P values <0.05 were regarded as significant. Where indicated, the results were presented as mean ±SD.

## Results and Discussion

### MTT screening assay

**Fig 2:**
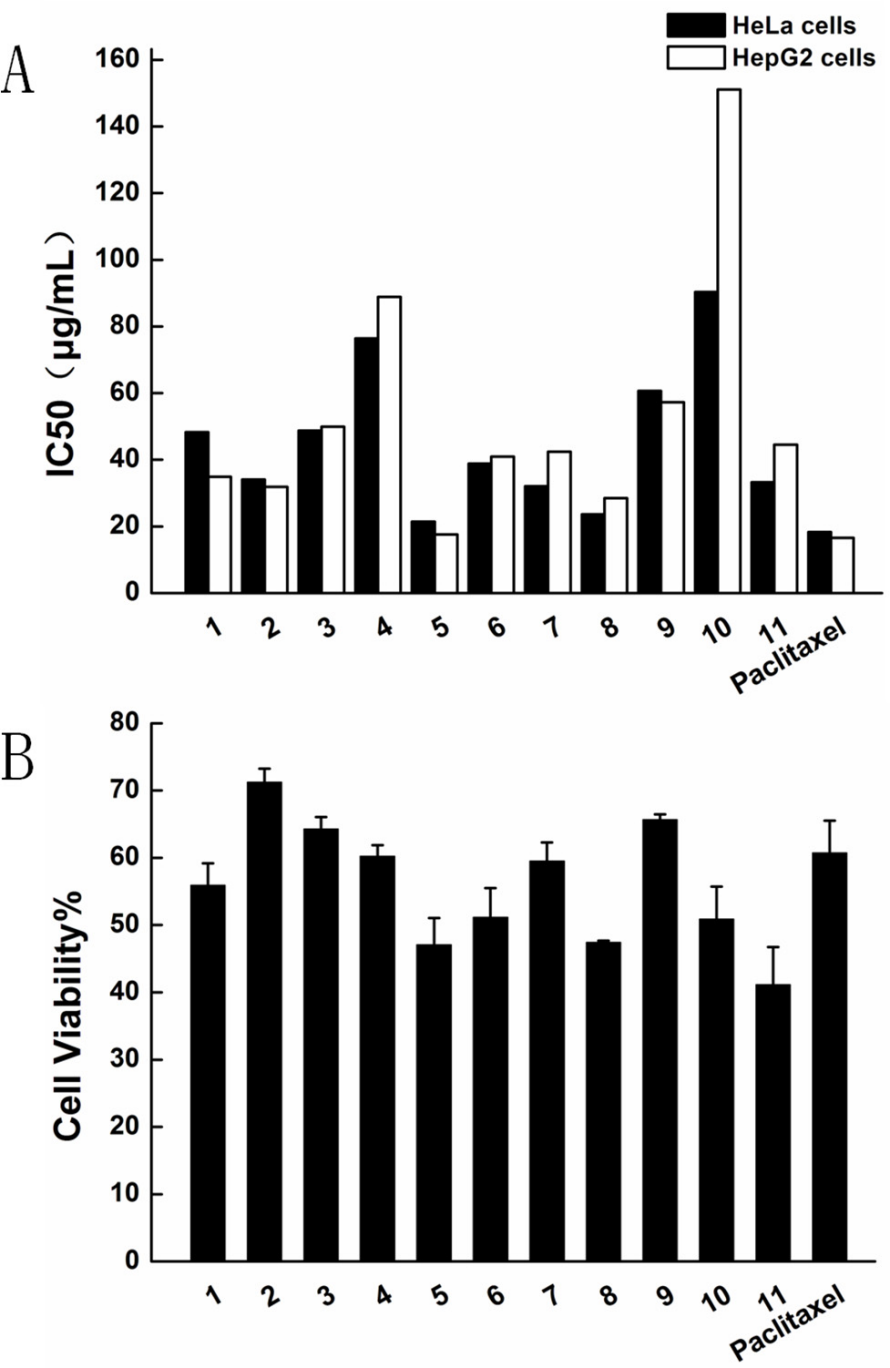
Cytotoxicity tests of compounds by MTT assay. Cells were treated with various compounds for 4h, and grown for another 20h. (A)IC50 of HeLa and HepG2 cells (B)HEK-293T cells Data represent the mean± standard deviation (n = 6).

Isoquinoline derivatives and α-methylene-γ-butyrolactones are bioactive compounds. For cytotoxicity assay, cancer cells (HeLa and HepG2 cells) and normal (HEK-293T) cells were treated with serial dilutions of 11 compounds. The cytotoxicity data (Fig 2A, Fig 2B) shows that they all had significant ability to inhibit cancer cells. Compound 2,5,11 had high cytotoxicity for cancer cells. Compound 2 was deemed the best agent because it had relative low cytotoxicity for HEK-293T cells but high cytotoxicity for HeLa and HepG2 cells. Compound 2 was, therefore, selected for further evaluation.

### Characterization of Tf-LP-Compound 2

The LP incorporation efficiency of Tf-PEG-DSPE was investigated by BCA Protein Assay, the results are shown in S1 Table and S1 Fig.

**Fig 3:**
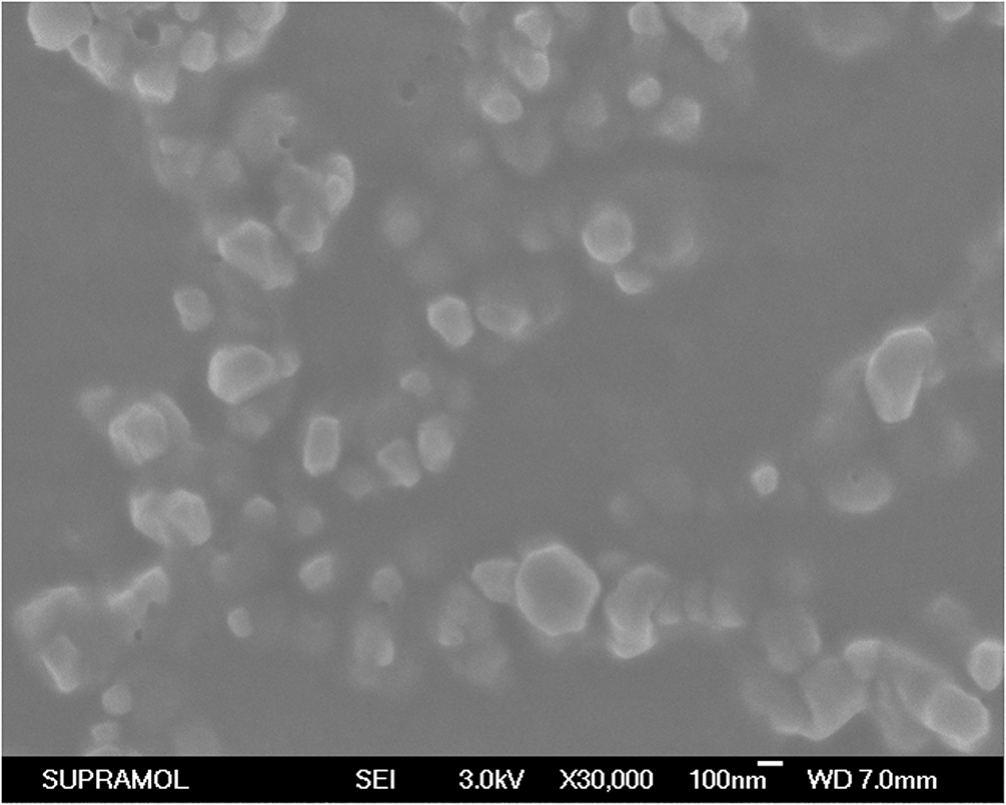
FE-SEM images of Tf-LP-Compound 2.

The morphology of the LPs was observed by FE-SEM and the results are shown in Fig 3. The FE-SEM image shows that most LPs were spherical particles with similar size and uniform dispersion. Other characteristics of the LPs before and after coupling of Tf were given in Table 1. Before addition of Tf, the particle size was 77.1±0.3nm. After addition of Tf, the particle size was increased to 144.5±1.7nm. The zeta potential of LPs was negative. There was not a significant change in zeta potential between the LP-Compound 2 and Tf-LP-Compound 2.The encapsulation efficiency of LPs was around 70–85%. Tf-LP-Compound 2 had somewhat lower encapsulation efficiency.

**Table 1:** Size, ζ-potential and Encapsulation efficiency of LPs before and after coupling of Tf at pH 7.4. Data are shown as means and standard deviation (n = 3).

Physical properties	Before addition of transferrin	After addition of transferrin
Size(nm)	77.1±0.3	144.5±1.7
ζ-potential(mV)	-6.4±4.7	-4.2±0.3
Encapsulation efficiency	82.0±1.7	73.7±1.6

### In vitro release studies

**Fig 4:**
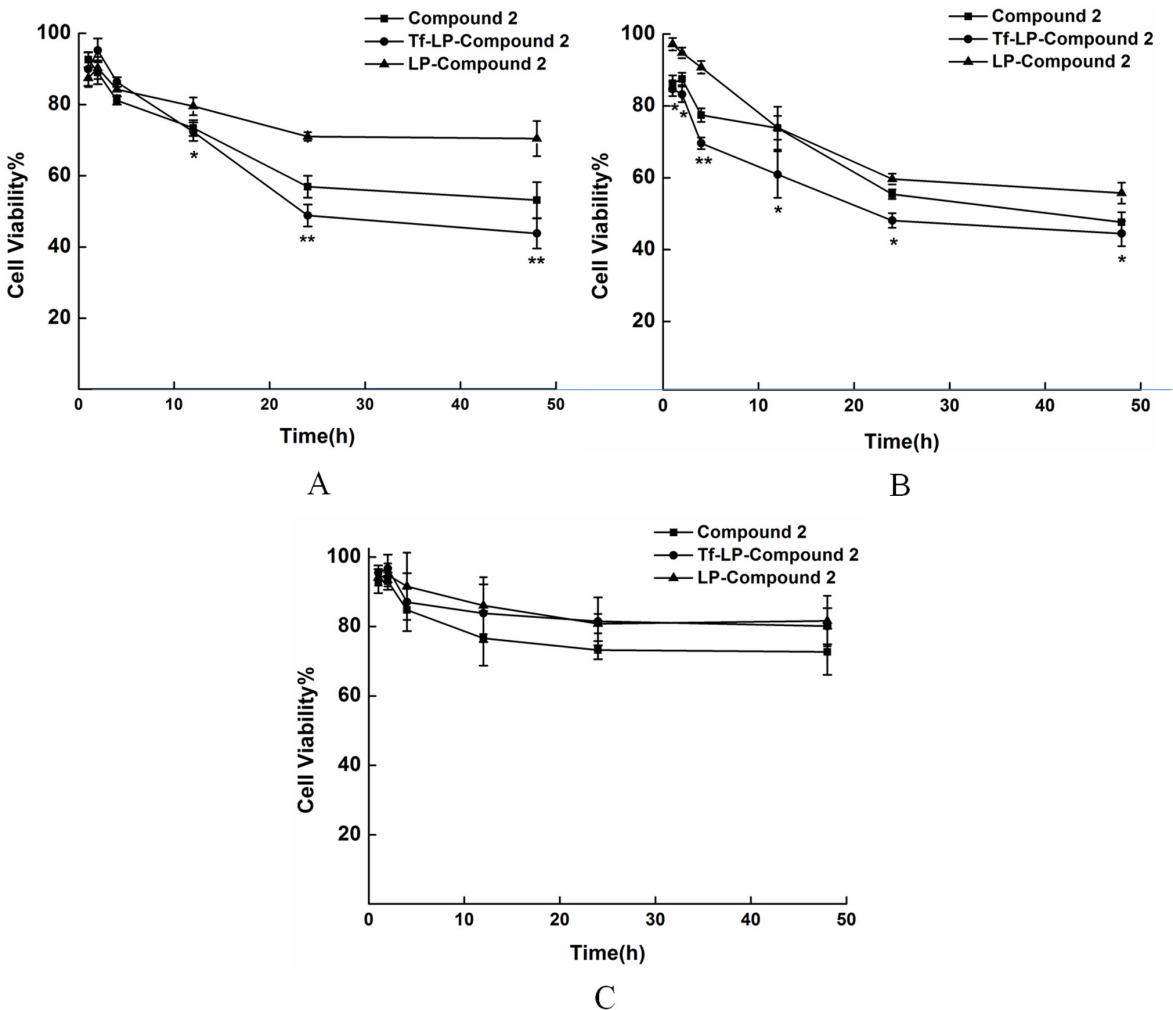
In vitro release of LP-Compound 2, Tf-LP-Compound 2 and free Compound 2. Drug release during 24 h incubation in PBS (0.5%v/v Tween 80) at 37 degree C, mean SD values (n = 3) are presented.

The in vitro release behaviors of the free Compound 2, LP-Compound 2, and Tf-LP-Compound 2, are shown in Fig 4. Release rates of the free Compound 2, LP-Compound 2 and Tf-LP-Compound 2, were measured at 37°C in PBS containing 0.5% Tween 80. Over a period of 24 h measurements were taken at 0, 0.5, 1, 2, 4, 8, 24 h. The release rate of the free drug was the highest. Meanwhile, the rate of drug release from Tf-LP-Compound 2 was slightly lower than that of LP-Compound 2. To access the stability of LP, we performed incubation in fetal bovine serum at 25 and 37 degree C for 48 h (S2 Fig). These results showed that Tf-LP-Compound 2 has substantial colloidal stability.

### Cytotoxicity assay of Tf-LP-Compound 2

**Fig 5:**
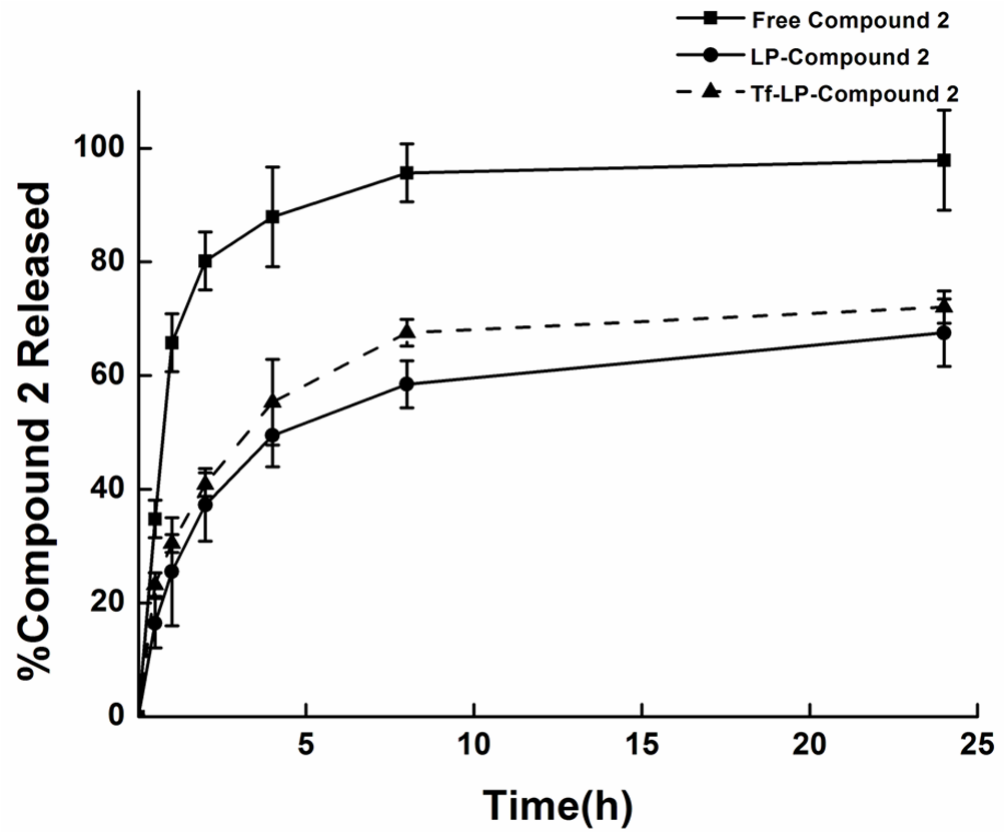
Cytotoxicity tests of various formulations in various cells. (A) HeLa cells (B) HepG2 cells (C) HEK-293T cells. Data represent the mean± standard deviation (n = 6) (*p<0.05, ** p<0.01: Tf-LP-Compound 2 versus LP-Compound 2).

In this study, HeLa, HepG2 and HEK-293T were selected to assess the cytotoxic effects of Compound 2, LP-Compound 2 and Tf-LP-Compound 2. Cell viabilities of the various formulations in HeLa and HepG2 cells indicated the order of Tf-LP-Compound 2< Compound 2<LP-Compound 2 (Fig 5A and 5B). The targeted Tf-LP-Compound 2 was significantly higher than that of LP-Compound 2. In HEK-293Tcells the cell viabilities of the various formulations indicated the order of Tf-LP-Compound 2≈ LP-Compound 2<Compound 2(Fig 5C) Tf-LP-Compound 2 showed high cytotoxicity for tumor cells and low cytotoxicity for normal cells, which is a favorable property for an anticancer drug. These results indicate that Tf was effective in promoting the internalization of LPs encapsulating Compound 2 to the target tumor cells.

### Mitochondrial membrane potential changes caused by Tf-LP-Compound 2

**Fig 6:**
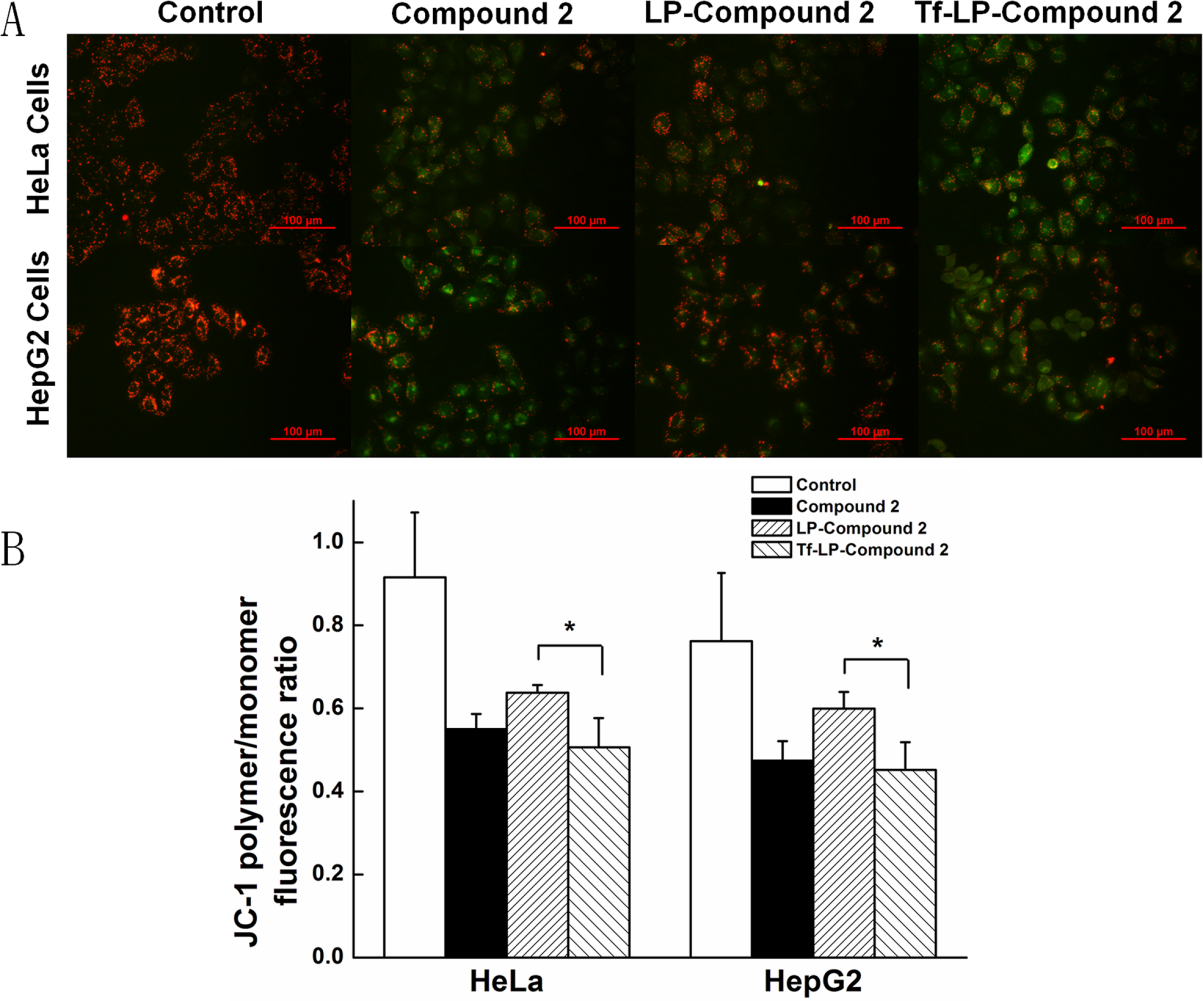
Fluorescence image of HeLa and HepG2 cells stained with JC-1. (A)Photograph showing JC-1 red, JC-1 green and merge image. (B)Numerical data were expressed in terms of the ratio of JC-1 aggregates to JC-1 monomers. Data are representative of three independent experiments and expressed as means ±SD, *p, 0.05, **p, 0.01 and ***p, 0.001 as compared with the control.

Red /green fluorescence ratio of JC-1 indicates the mitochondrial membrane potential (ΔΨm). Results obtained indicated that cancer cells (HeLa and HepG2 cells) treated with different formulations of LPs and free Compound 2 induced a strong green fluorescence (Fig 6A). The merge images of JC-1 red and green treated with Tf-LP-Compound 2 and free Compound 2 showed that majority of cells expressed strong green fluorescence, indicating potent apoptotic activity of Compound 2. As shown from quantitative data, the Red/Green-fluorescence ratio of Tf-LP-Compound 2 decreased from 0.92±0.16 to 0.51±0.07 in HeLa cells (from 0.76±0.16 to 0.45±0.07 in HepG2 cells). This was significantly different from LP-Compound 2 (Fig 6B). Furthermore, ROS generation was assessed at 12 h after LPs treatment. ROS generation in the cells treated with free Compound 2 and Tf-LP-Compound 2 was higher than LP-Compound 2(S3 Fig). Increased ROS generation is known to be involved in the induction of apoptosis through various pathways.

### Cellular uptake studies of Tf-LP-Compound 2 on HeLa cells

**Fig 7:**
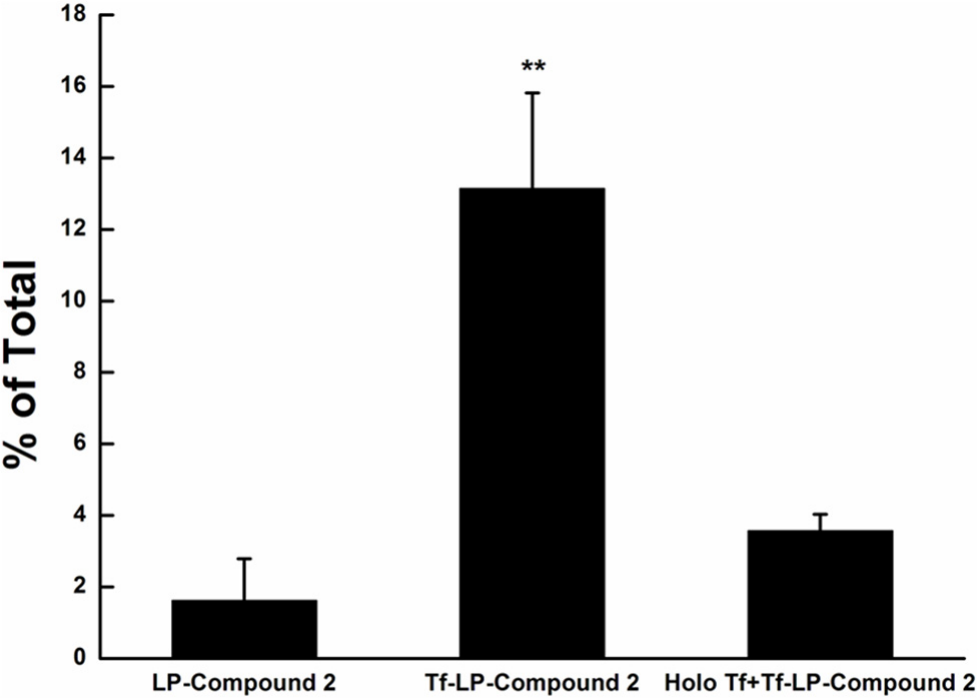
Cellular uptake of LP-Compound 2 and Tf-LP-Compound 2. Flow cytometry after incubation of LP-Compound 2, Tf-LP-Compound 2 and Tf-LP-Compound 2 with holo Tf. Data represent the mean± standard deviation (n = 3)(**p<0.01 vs LP-Compound 2).

Flow cytometry was used to quantify the LPs uptake by HeLa cells for LP-Compound 2, Tf-LP-Compound 2 and Tf-LP-Compound 2 with holo-Tf as shown in Fig 7. The fraction of NBD and rhodamine double positive cells incubated with LP-Compound 2 was 1.61%. For Tf-LP-Compound 2, the fraction was 13.14%. In competitive binding assays, the uptake from Tf-LP-Compound 2 was inhibited by 73% through the prior addition of 10 nM holo human Tf. These results demonstrated that the increased efficacy of Tf-LPs was due to TfR targeting. The addition of 10 nM holo human Tf to the incubation medium decreased compound uptake. The efficacy of this system is unlikely to be affected by the presence free Tf in vivo because most Tf in circulation is iron-free.

### Confocal microscopy evaluation of Tf-LP-compound 2 cellular uptake

**Fig 8:**
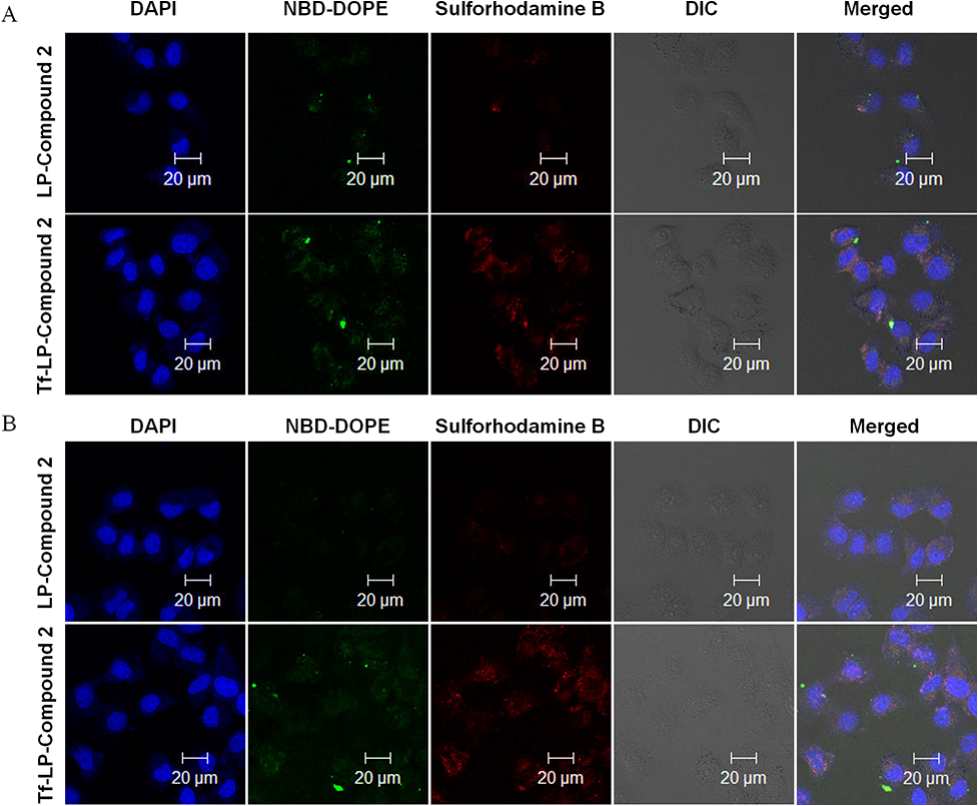
Intracellular localization of LP-Compound 2 and Tf-LP-Compound 2. Confocal microscopy analysis after 4 h of incubation of LP-Compound 2 and Tf-LP-Compound 2 at 37°C with HeLa cells (A) and HepG2 cells (B). NBD-DOPE fluorescence is shown in green, sulforhodamine B is shown in red, and DAPI nuclear stain is shown in blue.

The cellular association of the LP-Compound 2 and Tf-LP-Compound 2 was further investigated by confocal microscopy (Fig 8). After a 4-h exposure, Compound 2 delivered by the Tf-LPs accumulated to significant extents in both HeLa (Fig 8A) and HepG2 cells (Fig 8B). For the non-targeted LPs, a relatively small amount of LP-delivered Compound 2 appeared in the cells. DAPI was used for visualization of the nucleus. Confocal microscopy analysis indicated that association of Tf targeted LPs to HeLa and HepG2 cells was much greater than that of non-targeted LPs.

Moreover, the confocal microscopy was used detect visual signs of cellular damage. Cells treated with Tf-LPs had a worse growth status compared with LPs. The results suggest that Tf-LPs are efficient delivery vehicles for Compound 2.

## Conclusions

We have identified, among 11 compounds, Compound 2, an isoquinoline derivative, as a potential anticancer agent based on its ability to inhibit tumor cell growth and its having low toxicity to normal cells. Furthermore, we have shown that this compound can be efficiently loaded into Tf-conjugated LPs for targeted delivery to tumor cells, which overexpress the transferrin receptor. We believe the data obtained support further development of Tf-LP-Compound 2 as an anticancer agent. In future work, we plan to do further characterization of Compound 2 by determining its IC_50_ values in additional tumor cell lines and evaluate the antitumor activity of Tf-LP-Compound 2 in murine tumor models.

## Supporting Information

S1 Fig Cellular uptake of LPs with various Tf-PEG-DSPE in HeLa cells. Influence of Tf-PEG-DSPE concentrations on cellular uptake of LP-Compound 2 and Tf-LP-Compound 2. Data represent the mean± standard deviation (n = 3) (** p<0.01 vs LPs). (TIFF)
(DOCX)

S2 Fig LP stability in serum. Expressed as percent of initial Compound 2 present during 48 h incubation in FBS (10% v/v, in PBS pH 7.4) at 25 and 37℃C, mean SD values (n = 3) are presented. (TIFF)
(DOCX)

S3 Fig ROS generation in HeLa and HepG2 cells. Photomicrographs showing intracellular ROS generation in HeLa and HepG2 cells induced by Tf-LP-Compound 2 after 12 h incubation. Photomicrographs were taken by florescence phase contrast microscope. (TIFF)
(DOCX)

S1 Table The Coupling efficiency of Tf. Data are shown as means and standard deviation (n **=** 3). (PDF)
(DOCX)
