# Supplementary material for: A Novel Isoquinoline Derivative Anticancer Agent and Its Targeted Delivery to Tumor Cells Using Transferrin-Conjugated Liposomes
Source: PLoS One. 2015 Aug 26;10(8):e0136649. doi: 10.1371/journal.pone.0136649 (PMC4550422; doi:10.1371/journal.pone.0136649)
Supplement: S1 Fig — Influence of Tf-PEG-DSPE concentrations on cellular uptake of LP-Compound 2 and Tf-LP-Compound 2. Data represent the mean± standard deviation (n = 3) (** p<0.01 vs LPs). (TIFF) (DOCX) [file pone.0136649.s001.docx]

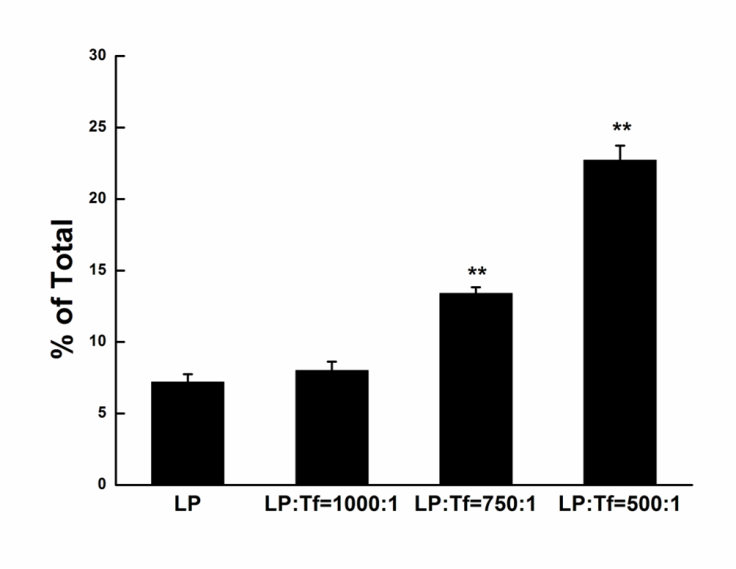


S1 Fig. Cellular uptake of LPs with various Tf-PEG-DSPE in HeLa cells. Influence of Tf-PEG-DSPE concentrations on cellular uptake of LP-Compound 2 and Tf-LP-Compound 2. Data represent the mean± standard deviation (n = 3) (** p<0.01 vs LPs).
