# Supplementary material for: A Novel Isoquinoline Derivative Anticancer Agent and Its Targeted Delivery to Tumor Cells Using Transferrin-Conjugated Liposomes
Source: PLoS One. 2015 Aug 26;10(8):e0136649. doi: 10.1371/journal.pone.0136649 (PMC4550422; doi:10.1371/journal.pone.0136649)
Supplement: S2 Fig — Expressed as percent of initial Compound 2 present during 48 h incubation in FBS (10% v/v, in PBS pH 7.4) at 25 and 37℃C, mean SD values (n = 3) are presented. (TIFF) (DOCX) [file pone.0136649.s002.docx]

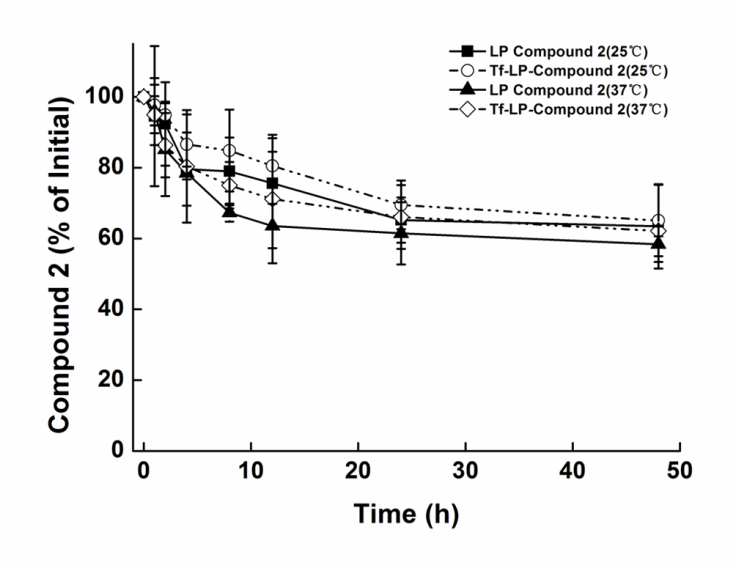


S2 Fig. LP stability in serum. Expressed as percent of initial Compound 2 present during 48 h incubation in FBS (10% v/v, in PBS pH 7.4) at 25 and 37 ℃, mean SD values (n = 3) are presented.
