# Supplementary material for: A Novel Isoquinoline Derivative Anticancer Agent and Its Targeted Delivery to Tumor Cells Using Transferrin-Conjugated Liposomes
Source: PLoS One. 2015 Aug 26;10(8):e0136649. doi: 10.1371/journal.pone.0136649 (PMC4550422; doi:10.1371/journal.pone.0136649)
Supplement: S3 Fig — Photomicrographs showing intracellular ROS generation in HeLa and HepG2 cells induced by Tf-LP-Compound 2 after 12 h incubation. Photomicrographs were taken by florescence phase contrast microscope. (TIFF) (DOCX) [file pone.0136649.s003.docx]

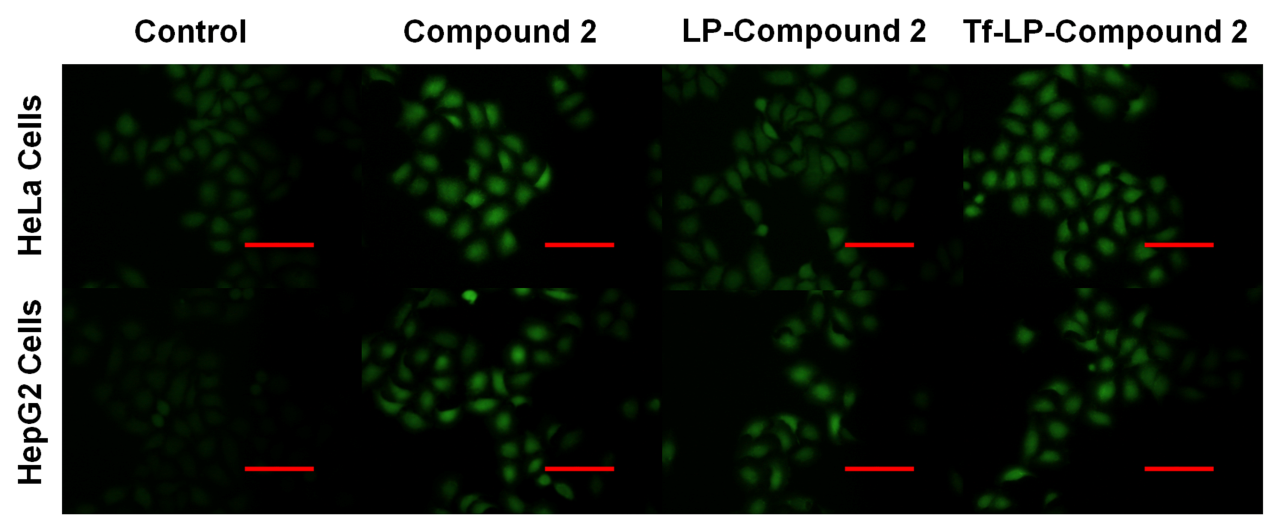
S3 Fig. ROS generation in HeLa and HepG2 cells. Photomicrographs showing intracellular ROS generation in HeLa and HepG2 cells induced by Tf-LP -Compound 2 after 12 h incubation. Photomicrographs were taken by florescence phase contrast microscope.
