# Supplementary material for: A Novel Isoquinoline Derivative Anticancer Agent and Its Targeted Delivery to Tumor Cells Using Transferrin-Conjugated Liposomes
Source: PLoS One. 2015 Aug 26;10(8):e0136649. doi: 10.1371/journal.pone.0136649 (PMC4550422; doi:10.1371/journal.pone.0136649)
Supplement: S1 Table — Data are shown as means and standard deviation (n = 3). (PDF) (DOCX) [file pone.0136649.s004.docx]

**S1 Table** The Coupling efficiency of Tf. Data are shown as means and standard deviation (n = 3).

| LP: Tf | 1000:1 | 750:1 | 500:1 |
| --- | --- | --- | --- |
| Coupling efficiency (%) | 14.65±2.68 | 31.80±0.24 | 33.44±0.41 |
